# Alignments of endocrine, anthropometric, and metabolic parameters in type 2 diabetes after intervention with an Okinawa-based Nordic diet

**DOI:** 10.29219/fnr.v62.1328

**Published:** 2018-03-14

**Authors:** Bodil Ohlsson, Gassan Darwiche, Bodil Roth, Peter Höglund

**Affiliations:** 1 Department of Internal Medicine, Skåne University Hospital, Lund University, Malmö, Sweden; 2 Department of Clinical Chemistry and Pharmacology, Skåne University Hospital, Lund University, Lund, Sweden

**Keywords:** adipokines, incretins, gut hormones, Okinawa-based Nordic diet, metabolic control

## Abstract

**Background:**

An Okinawa-based Nordic diet with moderately low carbohydrate content and high fat and protein content has been shown to improve anthropometry and metabolism in type 2 diabetes.

**Objective:**

The objectives of this study were to measure plasma or serum levels of hormones regulating energy metabolism and metabolic control, that is, cholecystokinin (CCK), Cortisol, C-peptide, ghrelin, glucagon, glucagon-like peptide-1 (GLP-1), glucose-dependent insulinotropic polypeptide (GIP), insulin, leptin, plasminogen activator inhibitor-1 (PAI-1), polypeptide YY (PYY), resistin, and visfatin after this diet intervention, and to determine partial correlations between hormonal levels and anthropometric and metabolic responses.

**Design:**

A total of 30 patients (17 women) with type 2 diabetes, mean age 57.5 ± 8.2 years, and body mass index (BMI) 29.9 ± 4.1 kg/m^2^ were served the diet for 12 weeks. Fasting hormones were measured by Luminex and enzyme–linked immunosorbent assay (ELISA) before study start and after 12 and 28 weeks, along with anthropometric and metabolic parameters.

**Result:**

The levels of CCK (*P* = 0.005), cortisol (*P* = 0.015), C-peptide (*P* = 0.022), glucagon (*P* = 0.003), GLP-1 (*P* = 0.013), GIP (*P* < 0.001), insulin (*P* = 0.004), leptin (*P* < 0.001), and PYY (*P* < 0.001) were lowered after dietary intervention. These reduced levels only remained for PYY at week 28 (*P* = 0.002), when also ghrelin (*P* = 0.012) and visfatin (*P* = 0.021) levels were reduced. Changes of glucose values correlated with changed levels of C-peptide and PYY (*P* < 0.001), insulin (*P =* 0.002), and PAI-1 (*P* = 0.009); changes of triglyceride values with changed levels of C-peptide, insulin, and PYY (*P* < 0.001) and PAI-1 (*P* = 0.005); changes of insulin resistance with changes of leptin levels (*P* = 0.003); and changes of BMI values with changed levels of C-peptide, insulin, and leptin (*P* < 0.001).

**Conclusions:**

Okinawa-based Nordic diet in type 2 diabetes has significant impact on the endocrine profile, which correlates with anthropometric and metabolic improvements.

High intake of plant-derived foods and a lower intake of red meat, meat products, sweets, salt, high-fat dairy, and refined grains are considered to be important features of a healthy diet (1). There is a growing interest in healthy dietary patterns, such as Mediterranean and Okinawan diets, to improve metabolism, inflammation, and cardiovascular health in the population (2). A modified Okinawan diet has been developed consisting of moderately low carbohydrate content and higher contents of fiber, fat, and protein, with food components suitable for the Nordic population (3). A single meal of this diet to healthy volunteers has been shown to attenuate the postprandial responses of glucose, C-peptide, insulin, and glucose-dependent insulinotropic polypeptide (GIP), leaving the secretion of adipokines, ghrelin, glucagon, glucagon-like peptide-1 (GLP-1), and plasminogen activator inhibitor-1 (PAI-1) unaffected (4). The increased postprandial satiety did not correlate with any hormonal changes (4).

The question remains as to how much of the responses after a single meal are reflected in long-term effects of dietary interventions. Both luminal carbohydrates and fat induce postprandial secretion of the incretins GIP and GLP-1, GIP secretion being more sensitive to carbohydrate stimulation and GLP-1 secretion being more sensitive to fat stimulation (5, 6). Apart from facilitating glucose-stimulated insulin secretion, GIP also has a role in obesity development through increased hydrolysis of circulating triacylglycerides, with subsequent re-esterification of free fatty acids into triacylglycerides in adipocytes (7). GLP-1 is the most potent incretin and it improves glucose homeostasis during meals by increasing insulin secretion and reducing food intake, gastrointestinal motility, secretion of digestive enzymes into the lumen, and glucagon secretion (8, 9). As GLP-1 reduces intestinal motility, this peptide mediates a better proximal fat absorption (5).

Cholecystokinin (CCK) is released in response to fat and protein, and it is an important hormone in the regulation of gastrointestinal motility and satiety (10). Polypeptide YY (PYY) is secreted in response to fat and protein intake and levels appear to be affected by acute exercise, adiposity, and composition of macronutrients (including fiber) and fatty acids from dietary fat (11). The most important effect of PYY is regulation of appetite and body weight, but recent research suggests that PYY also has an impact on beta cell mass, thereby participating in glucose homeostasis (12). Ghrelin plays a role in body energy metabolism and its concentration is greatest in the fasting state, to be suppressed in response to meal intake (13).

Adipokines, that is, leptin, resistin, and visfatin, are hormones released from the adipose tissue and have a central role in the control of energy metabolism, regulation of glucose and lipid metabolism, and insulin sensitivity (14). These hormones are supposed to be involved in the development of obesity, diabetes, inflammation, auto-immunity, and metabolic syndromes (15). Elevated levels of PAI-1 form a link between obesity, insulin resistance, and the risk of cardiovascular events (16). Cortisol is assumed to be involved in the development of metabolic syndrome and type 2 diabetes (17).

Our hypothesis was that the Okinawa-based Nordic diet influences the hormone secretion in type 2 diabetes, in alignment with changes in metabolic and anthropometric parameters. The diet was given to patients with type 2 diabetes for 12 weeks, with a follow-up after another 16 weeks. Blood samples and clinical examinations were taken before the study started, and after 12 and 28 weeks. The beneficial impact on metabolic and anthropometric parameters has been presented in a previous report (3). The primary objective of the present study was to assess changes in plasma levels of hormones regulating satiety and metabolic control, that is, CCK, Cortisol, C-peptide, ghrelin, glucagon, GLP-1, GIP, insulin, leptin, PAI-1, PYY, resistin, and visfatin, during and after a dietary intervention. The secondary objectives were to determine partial correlations between hormonal levels and metabolic responses, blood pressure, body mass index (BMI), weight, and waist circumference.

## Methods and materials

The subjects were treated according to the Declaration of Helsinki and the study was approved by the Regional Ethics Review Board at Lund University (2014/460). All subjects gave their written, informed consent before participating in the study which was monitored by an external monitor and registered at ClinicalTrials.gov data base (NCT02405806).

### Study population

Patients with type 2 diabetes, independently of BMI or anti-diabetic treatment regimen, aged between 18 and 70 years, were recruited from diabetes patients at a primary health care center in the southernmost district of Sweden.Patients were to have both parents born in Scandinavia, to avoid possible influence of ethnicity on the study results. Overall, exclusion criteria were severe food allergy, and severe heart, pulmonary, cardiovascular, malignant, or psychiatric diseases. Patients with type 1 diabetes, severe liver insufficiency, defined as spontaneous international normalized ratio (INR) > 1.1, or severe renal insufficiency, defined as estimated glomerular filtration rate (eGFR) < 30 mL/min/1.73m^2^, as well as patients with a prior major gastrointestinal surgery were excluded. Patients with known alcohol and drug abuse were not considered for inclusion. Participants were initially informed through a mail of the project design and purpose (Supplementary Fig. 1). One week later, all the patients were contacted by phone by one of the three investigators (BO and GD, physicians, or the nutritionist).

### Study design

The trial was a clinical prospective interventional study with the patients being their own controls, performed at Skåne University Hospital, Malmö, Sweden, and conducted for 12 weeks with an Okinawa-based Nordic diet, followed by a clinical follow-up after 16 weeks with unrestricted diets. A detailed description of the study design, diet components, and methodology has recently been published in a separate publication (3). Briefly, all tests were performed under standardized conditions and stable temperatures. The study data consisting of blood sampling, assessments of anthropometric data, and completion of questionnaires were obtained at three separate visits: (1) at study start before introduction of the diet; (2) after 12 weeks on the Okinawa-based Nordic diet; and (3) after 16 weeks on unrestricted diet. In addition, two visits after 2 and 6 weeks were performed to assess anthropometric data and complete a protocol to check for compliance (Supplementary Fig. 1). All participants were instructed by a nutritionist on how to prepare their breakfast, based on the data from the nutrition questionnaire. Food for lunch, dinner, and snacks was delivered home in a cooler bag three times a week, free of charge, along with written information and recipes for meal preparation. The participants were in close contact with a nutritionist throughout the study and compliance was registered. The participants were encouraged to maintain their regular physical activity habits throughout the intervention. Blood samples were collected through an intravenous catheter after a 10-h fast. Metabolic parameters were analyzed at once, and plasma and serum were harvested and stored at −80°C until analyzed for hormonal concentrations. The study started on 2 February 2015 and ended on 18 September 2015.

### Diets

The diet is based on the traditional Okinawan diet (1) but modified to suit the taste and food components suitable for the Nordic population (3). The meal composition is consistent with moderately low carbohydrate content, one of four nutritional recommendations from the Swedish National Food Agency for patients with diabetes (18). These recommendations are in line with international recommendations (American Diabetes Association [ADA], European Association for the Study of Diabetes [EASD]). At the same time, the contents of fiber, fat, and protein are increased, which lead to a bigger meal demanding more mastication and prolonged meal intake (4). The food is based on traditional Nordic raw food, for example, whole grains, vegetables, legumes, root crops, fatty fish, fruits, berries, and nuts, with minimal industrial processing. Furthermore, the amount of dairy products, red meat, and processed meat, as well as sugar and white flour was limited to have a diet with low glycemic index (GI). The diet has a good nutritional supply including a mean calorie intake of around 1,900 kcal/day, which is slightly lower compared with a traditional diet. The participants were allowed to eat three meals a day, including breakfast, lunch, and dinner, and two snacks between meals consisting of a variety of fruits, berries, and seeds. Organic food items were preferred whenever possible. During cravings, the subjects were instructed to eat a third snack (e.g. carrots, boiled eggs, mackerel in tomato sauce, or cottage cheese with berries) to avoid eating fast carbohydrates. Raw vegetables or green salad were to be ingested with the main meals – 100 g at breakfast and 150 g at lunch and dinner, respectively. The participants were instructed always to start with the vegetables and to eat slowly. Nutrition information is given in Supplementary Table 1. Darwiche et al. (3) have described the details of the food composition.

The meals were planned at the kitchen of Igelösa Life Science Lab (Lund University) and delivered to the subjects regularly free of charge, along with written information and recipes for meal preparation. Two breakfast alternatives were ingested which consisted of porridge or fermented milk in combination with bread, depending on their ordinary breakfast, and the subjects had to buy the breakfast themselves.

No dietary supplements such as fish oil, probiotics, or multivitamin drugs were allowed to be introduced during the study period. One visit to a restaurant or for another diet per week was allowed. Journeys or a stay during a longer time period at another place had to be discussed with the investigators. Maximal intake of alcoholic beverages was set at 30 g ethanol/week, using the following formula: volume% × mL volume/100 × 0.8.

During the study period, the nutritionist met the participants at baseline, and at 2, 6, 12, and 28 weeks afterward (Supplementary Fig. 1). At week 2, a dietary follow-up was conducted with the ability to adjust the food composition. Furthermore, the nutritionist emailed information to all participants weekly, and they could reach the nutritionist by email and telephone calls when needed, to support the subjects and enhance compliance as much as possible. They also had the opportunity to provide written feedback to the nutritionist using the returned cooler bag. Participants completed a nutrition questionnaire which was collected at study start, and at week 12 and 28, and also completed a food diary during the 12 intervention weeks, from which they received feedback by the nutritionist. The participants had a good adherence to the diet, as described in detail previously (3).

### Assessment of clinical variables and anthropometry

The investigations took place under similar conditions by two clinically experienced physicians (BO and GD) at baseline and at 2, 6, 12, and 28 weeks afterward. Physical examination included cardiopulmonary, abdominal, and neurological examinations as well as measurements of blood pressure, pulse, respiratory rate, weight, height, waist circumference, and assessment of BMI. Blood pressure was measured in the supine position. Weight was measured in patients wearing light clothes without shoes. Normal weight was defined as BMI < 25 kg/m^2^, overweight as BMI ≥ 25 kg/m^2^ but <30 kg/m^2^, and obesity as BMI ≥ 30 kg/m^2^ (19). Waist circumference was measured, midway between the lower border of the rib cage and the superior border of the iliac crest (20). Diabetic complications were registered including autonomic neuropathy (sexual dysfunction, profound sweating, and orthostatic blood pressure), gastrointestinal dysmotility (based on motility examination), levels of albuminuria (measured as albumin/creatinin ratio), macroangiopathy, peripheral neuropathy (examined by patellar and achilles tendon reflexes, vibration sense, and monofilament), and retinopathy (based on fundus photography). The study questionnaire contained questions about socioeconomic factors, medical history, and lifestyle habits, and was completed at baseline and at week 12 and week 28. A more simple protocol was completed at baseline including information on whether the participants already were on ongoing weight-reducing diet; intake of dietary supplements, vitamins, and probiotics; or food allergy. Another protocol including information about changes in medication, physical activity, or routines, as well as any extraordinary events of daily life during the study time was completed at 2, 6, 12, and 28 weeks afterward (Supplementary Fig. 1).

### Blood sampling and chemistry analyses

All samples consisted of whole blood drained into ethylenediaminetetra-acetic acid (EDTA) glass tubes (BD Microtainer, Franklin Lakes, New Jersey, USA) or serum separation tubes (SST) with coagulation activator and gel (BD Microtainer). Blood was centrifuged at 3,000 rcf for 10 min, and plasma and serum were immediately cooled and stored in −80^°^C until analyzed for later hormonal analyses. Cortisol, C-peptide, and insulin in serum; glycated hemoglobin A1c (HbA1c) in blood; and glucose, triglycerides, cholesterol, high-density lipoprotein (HDL), and low-density lipoprotein (LDL) in plasma were analyzed by standard methods in the Department of Clinical Chemistry. Homeostasis model assessment for insulin resistance (HOMA2-IR) was calculated using the HOMA2 calculator version 2.2.3 (21), after exclusion of extreme values of fasting plasma glucose and serum C-peptide.

### Hormonal analyses

The Luminex analyses were performed in all samples at the same time within 9 months. Human diabetes 10-plex panel (Bio-Plex Pro™ Human Diabetes Immunoassay control no 5029560-1 and 5040782, Bio-Rad Laboratories, CA, USA) was performed on the Luminex-200 (Luminex xMAP, Bio-Rad Laboratories) and data were analyzed using Bio-Plex Manager software 6.0 (Bio-Rad Laboratories). Hormones (pg/mL) measured were ghrelin, glucagon, GLP-1, GIP, leptin, PAI-1, resistin, and visfatin.

Analyses were performed blinded according to the manufacturer’s instructions. Briefly, samples were diluted 1:4 and incubated with magnetic beads coupled to specific capture antibodies. After a series of washes in a magnetic wash station (Bio-Plex Handled magnetic washer, 171020100, Bio-Rad Laboratories), biotinylated detection antibodies were added to form a sandwich complex. The final detection complex was created with the addition of streptavidin-phycoerythrin (SA-PE) conjugate. Absolute concentrations were measured from standards provided with the kit. Each run included controls with high and low concentrations for each biomarker, and a blank sample. All samples were analyzed in duplicate and the concentration of hormones bound to each bead was proportional to the median fluorescence intensity (MFI) of reporter signal. Standard curves were calculated with nonlinear regression type 5 parameter logistic. Inter-assay and intra-assay coefficients of variation (cv) for controls are presented in Supplementary Table 2.

Human serum CCK and PYY were analyzed with a commercial competitive inhibition enzyme–linked immunosorbent assay (ELISA) (Cloud-Clone Corp. Houston, Texas, USA, CEB802 Hu and CEB067 Hu, respectively) kit according to the manufacturer’s instructions. Standards (0, 12.35, 37.04, 111.11, 333.33, and 1,000 pg/mL), serum sample (50 uL/well), and biotin-labelled CCK and PYY, respectively, were pipetted in duplicates into plates pre-coated with a monoclonal antibody specific to CCK or PYY. The unbound conjugate was washed off and avidin conjugated to horseradish peroxidase (HRP) was added. After a second wash and addition of the 3,3′,5,5′-tetramethylbenzidine (TMB) substrate solution, the intensity of color was measured at 450 nm. CCK or PYY in the samples were reversed proportional to the amount of bound HRP. CCK and PYY concentrations in each sample were interpolated from the standard curve. Intra-assay and inter-assay were CV% <10% and <12%, respectively, for both CCK and PYY.

## Statistical methods

Two hypotheses were raised: (1) Intervention with an Okinawa-based Nordic diet affects the concentration of adipokines, CCK, cortisol, C-peptide, ghrelin, glucagon, incretins, insulin, PAI-1, and PYY, and (2) the hormone concentration correlates with metabolic responses and changes in blood pressure, BMI, weight, and waist circumference.

A power analysis was performed *a priori* based on a previous unpublished pilot study, and we determined that nine subjects were required to demonstrate that a weight reduction of 5–10%, accompanied by lower blood glucose, lower blood pressure, and improved lipid levels, would lead to clinically significant differences in metabolic parameters to reduce cardiovascular risk factors with 80% power at 5% significance level, as postulated in a previous study (22). The variable demanding most subjects to be able to discover was, what we expected, the diastolic blood pressure. We determined that we needed 18 subjects to demonstrate clinically significant differences in diastolic pressure with 80% power at 5% significance level. To be able to compensate for disappearance, we planned to recruit 25–35 subjects. Two of the recruited subjects interrupted the study at 6 weeks on diet, the time point that was considered as end of the intervention, and data collected at that time point was calculated together with data from subjects with 12 weeks of intervention.

We tested the hypotheses with linear mixed effect models to analyze continuous variables, with random intercept and unstructured co-variances for repeated measures within a subject, with visits as nominal fixed effect, using baseline as reference. We assumed that missing observations were unrelated to the observed value, that is, missing at random. In these analyses, predicted mean values and their 95% confidence limits are presented, together with estimates of changes from baseline and 95% confidence limits and *P*-values for the changes between baseline and week 12 and week 28. Descriptive statistics are given as means and standard deviations for continuous variables and as counts or frequencies for categorical variables. Since we had more than one observation from each subject, partial Spearman’s correlations, controlling for subject, were calculated. Statistics were done using MATLAB R2015a (Mathworks Inc.). *P* < 0.05 was considered statistically significant in the calculated changes of values. Due to multiple testing between each hormone and anthropometric and metabolic factors, *P* < 0.01 was considered statistically significant in the partial correlations.

## Results

### Basal characteristics

In total, 45 patients with diabetes were randomly selected after consideration of inclusion and exclusion criteria. Of these, 30 patients (67%) (17 women), mean age 57.5 ± 8.2 (range 40–67) years, accepted the invitation. Reasons for not being included were unwillingness to participate (*n* = 11), late autoimmune diabetes in adult (LADA) (*n* = 1), a history of gastric by-pass surgery (*n* = 1), pregnancy (*n* = 1), or already on a diet (*n* = 1) (Supplementary Fig. 1). The mean diabetes duration was 10.4 ± 7.6 (range 1–30) years. The treatment was metformin (40%), metformin in combination with insulin (27%), insulin solely (13%), metformin in combination with sulfonylurea (7%), diet solely (7%), sulfonylurea (3%), or dipeptidyl peptidase-4 (DPP 4) inhibitors (3%). The most common secondary complication to the disease was autonomic neuropathy and/or peripheral neuropathy (30%), retinopathy (27%), and nephropathy and macroangiopathy (17% in both). Only one patient had a verified gastroparesis.

Sixteen percent had only completed primary school, 57% had completed high school, and 27% had a higher degree of education. The majority, 67%, were employed, whereas 17% were retired. The rest of the patients were on sick leave or unemployed. Twenty of the patients were married or cohabitated, whereas six were divorced or widow/widower, and four were living alone. Smoking and usage of snuff both occurred in 23% of the patients. Half of the patients drank alcoholic beverages once a month or less, 27% drank alcohol 2–4 times a month, 13% drank alcohol 2–3 times a week, and 10% were teetotalers. A moderate but sporadic physical exercise during leisure time was most common (53%), followed by a moderately regular exercise (27%), regular exercise and training (13%), and sedentary leisure time (7%). Antihypertensive medication was prescribed in 63% of patients and lipid-lowering medication in 47%.

### Changes in anthropometric and metabolic parameters

The mean BMI at inclusion was 29.9 ± 4.1 kg/m^2^, and 50% of the patients (*n* = 15) were obese. During the interventional period of 12 weeks, the body weight was reduced (*P* < 0.001), accompanied by a reduction of BMI (*P* < 0.001) and waist circumference (*P* < 0.001), calculated by linear mixed model (Table 1). At week 12, only 12 patients were classified as obese. At week 28, the mean weight, BMI, and waist circumference remained significantly lower than at baseline (*P* < 0.001) (Table 1). Both systolic and diastolic blood pressures were decreased at week 12, and the diastolic blood pressure remained lower also at week 28, compared with baseline (Table 1). Blood levels of HbA1c, and plasma levels of glucose, triglycerides, cholesterol, and LDL were decreased during the dietary intervention, but only the HbA1c levels were still decreased at week 28, when also the HDL levels were increased, calculated by linear mixed model (Table 2).

**Table 1:** Anthropometric parameters in type 2 diabetes before and after a 12-week Okinawa-based Nordic diet intervention

Variable	Mean value	95% CI		Mean change	95% CI		*P*
	
		Lower	Upper		Lower	Upper	
Weight (kg)							
Baseline	89.8	84.5	95.1		
Week 12	83.6	78.1	89.0	−6.20	−7.61	−4.78	<0.001
Week 28	85.4	79.7	91.1	−4.40	−6.57	−2.24	<0.001
BMI (kg/m^2^)
Baseline	29.9	28.4	31.3		
Week 12	27.8	26.3	29.4	−2.05	−.52	−1.57	<0.001
Week 28	28.4	26.8	30.0	−1.47	−2.13	−0.82	<0.001
Waist circumference (cm)							
Baseline	107.3	103.4	111.2	
Week 12	100.3	96.1	104.4	−7.02	−8.62	−5.42	<0.001
Week 28	101.7	97.6	105.9	−5.54	−7.11	−3.96	<0.001
Systolic blood pressure (mmHg)
Baseline	140.17	134.72	145.61		
Week 12	130.55	124.39	136.71	−9.62	−13.30	−5.93	<0.001
Week 28	139.74	133.84	145.65	−0.42	−3.65	2.81	0.796
Diastolic blood pressure (mmHg)
Baseline	82.33	78.73	85.93		
Week 12	74.88	71.03	78.73	−2.70	−3.57	−1.84	<0.001
Week 28	78.74	74.76	82.72	−1.75	−2.78	−0.72	0.001

The mean values and mean changes and 95% confidence interval (CI) with lower and upper limits are presented for anthropometric parameters at inclusion (baseline) (*n* = 30), 12 weeks after diet intervention (*n* = 30), and 16 weeks after the end of diet intervention (week 28) (*n* = 23). Linear mixed model. Comparisons were made between baseline and week 12 and week 28. *P* < 0.05 was considered statistically significant.

**Table 2:** Circulating metabolic biomarkers in type 2 diabetes before and after a 12-week Okinawa-based Nordic diet intervention

Variable	Mean value	95% CI		Mean change	95% CI		*P*
	
		Lower	Upper		Lower	Upper	
Fasting Glucose (mmol/L)							
Baseline	9.71	8.54	10.87		
Week 12	7.91	6.55	9.27	−1.80	−2.63	−0.96	<0.001
Week 28	9.28	7.71	10.85	−0.42	−1.58	0.73	0.466
HbA1c (mmol/mol)
Baseline	61.57	56.42	66.72		
Week 12	49.20	44.02	54.38	−12.37	−16.40	−8.33	<0.001
Week 28	54.36	48.83	59.90	−7.20	−11.68	−2.72	0.002
HOMA2-IR (U)
Baseline	3.00	2.50	3.51		
Week 2	2.53	1.96	3.09	−0.48	−0.85	−0.11	0.012
Week 12	2.37	1.82	2.91	−0.64	−0.98	−0.30	<0.001
Week 28	2.61	2.05	3.16	−0.40	−0.75	−0.05	0.025
Triglycerides (nmol/L)
Baseline	1.79	1.41	2.16		
Week 12	1.49	1.09	1.89	−0.30	−0.52	−0.08	0.009
Week 28	1.96	1.46	2.46	0.17	−0.20	0.54	0.367
Cholesterol (mmol/L)
Baseline	4.65	4.36	4.95		
Week 12	4.22	3.87	4.57	−0.44	−0.69	−0.18	0.001
Week 28	4.71	4.37	5.05	0.06	−0.18	0.30	0.636
HDL (mmol/L)
Baseline	1.22	1.10	1.35		
Week 12	1.19	1.05	1.32	−0.04	−0.10	0.03	0.267
Week 28	1.34	1.20	1.47	0.11	0.04	0.18	0.003
LDL (mmol/L)
Baseline	2.92	2.62	3.22		
Week 12	2.68	2.33	3.03	−0.24	−0.48	−0.01	0.041
Week 28	2.82	2.49	3.16	−0.10	−0.30	0.11	0.356

The mean values and mean changes and 95% confidence interval (CI) with lower and upper limits are presented for fasting blood levels of glycated hemoglobin A1c (HbA1c) and fasting plasma levels of glucose, triglycerides, cholesterol, high-density lipoprotein (HDL), and low-density lipoprotein (LDL) at inclusion (baseline) (*n* = 30), 12 weeks after diet intervention (*n* = 30), and 16 weeks after the end of diet intervention (week 28) (*n* = 23). Insulin resistance was measured by HOMA2-IR (21). Linear mixed model. Comparisons were made between baseline and week 12 and week 28. *P* < 0.05 was considered statistically significant.

Only two patients had diet management as the sole treatment for diabetes. In 15 subjects, the anti-diabetes medication was gradually reduced during dietary intervention, and 2 subjects, 1 on both oral hypoglycemic agents and insulin, had their medication cancelled. Of the other 12 subjects with insulin treatment, 3 had their insulin therapy cancelled and another 8 had their insulin doses reduced.

Many of the patients had irregular meal habits before inclusion in the study, and often omitted breakfast and/or lunch. At the 28-week follow-up, most patients had kept their regular meal order with the same intake of breakfast as during the intervention, and with an increased intake of vegetables and legumes. The composition of lunch and dinner was partly kept. Seven patients were lost at follow-up because of depression (*n* = 2), family-related problems (*n* = 2), unwillingness to show up (*n* = 2), or work-related time constraints (*n* = 1) (Supplementary Fig. 1).

### Changes in hormonal concentrations

At week 12, the plasma or serum levels of CCK (*P* = 0.005), cortisol (*P* = 0.015), C-peptide (*P* = 0.022), glucagon (*P* = 0.003), GLP-1 (*P* = 0.013), GIP (*P* < 0.001), insulin (*P* = 0.004), leptin (*P* < 0.001), and PYY (*P* < 0.001) were significantly lowered compared with baseline, calculated by linear mixed model. There was a non-significant decrease of PAI-1 levels (*P* = 0.082) (Table 3). At week 28, the plasma levels of PYY (*P* = 0.002) were still lowered, in addition to the levels of ghrelin (*P* = 0.012) and visfatin (*P* = 0.021). Serum levels of insulin (*P* = 0.089) and plasma levels of resistin (*P* = 0.082) were non-significantly lowered at week 28 (Table 3).

**Table 3:** Circulating hormonal levels in type 2 diabetes before and after a 12-week Okinawa-based Nordic diet intervention

	Mean	95% CI		Mean change	95% CI		*P*
	
		Lower	Upper		Lower	Upper	
CCK (pg/mL; serum)							
Baseline	27	21	32		
Week 12	20	14	26	−7	−11	−2	0.005
Week 28	23	16	30	−3	−9	3	0.267
Cortisol (nmol/L; serum)
Baseline	367	337	397		
Week 12	325	290	360	−41	−74	−8	0.015
Week 28	348	315	382	−18	−50	13	0.249
C-peptide (nmol/L; serum)
Baseline	0.99	0.82	1.17		
Week 12	0.88	0.70	1.05	−0.11	−0.21	−1.02	0.022
Week 28	0.88	0.68	1.09	−0.11	−0.24	0.03	0.131
Ghrelin (pg/mL; plasma)
Baseline	852	608	1094		
Week 12	798	555	1041	−53	−139	32	0.219
Week 28	728	481	975	−123	−219	−28	0.012
Glucagon (pg/mL; plasma)
Baseline	1.93	1.29	2.58		
Week 12	1.28	0.63	1.92	−0.66	−1.09	−0.23	0.003
Week 28	1.74	1.06	2.42	−0.19	−0.67	0.29	0.426
GLP-1 (pg/mL; plasma)
Baseline	2.66	1.79	3.54		
Week 12	1.78	0.91	2.66	−0.88	−1.56	−0.19	0.013
Week 28	2.36	1.43	3.30	−-0.30	−1.08	0.48	0.477
GIP (pg/mL; plasma)
Baseline	78	57	99		
Week 12	55	34	76	−23	−36	−10	<0.001
Week 28	75	52	98	−3	−18	12	0.692
Insulin (mIU/L; serum)
Baseline	15.53	12.67	18.40		
Week 12	11.67	8.80	14.53	−3.87	−6.43	−1.30	0.004
Week 28	12.96	9.72	16.19	−2.58	−5.55	0.40	0.089
Leptin (pg/mL; plasma)
Baseline	9,404	587	1,294		
Week 12	5,850	199	971	−3,554	−5,328	−1,779	<0.001
Week 28	8,553	491	1,219	−851	−2,078	376	0.171
PAI-1 (pg/mL; plasma)
Baseline	72,434	58,869	85,998		
Week 12	66,413	52,402	80,425	−6,020	−12,814	774	0.082
Week 28	71,810	56,705	86,915	−623	−9,455	8,209	0.889
PYY (pg/mL; plasma)
Baseline	1,937	1,802	2,073		
Week 12	1,510	1,360	1,660	−427	−572	−282	<0.001
Week 28	1,673	1,502	1,844	−264	−431	−97	0.002
Resistin (pg/mL; plasma)
Baseline	5,234	3,904	6,565		
Week 12	5,043	3,705	6,382	−191	−1,118	736	0.683
Week 28	4,325	2,915	5,736	−909	−1,938	119	0.082
Visfatin (pg/mL; plasma)
Baseline	998	732	1,264		
Week 12	1,115	816	1,415	117	−78	312	0.235
Week 28	812	535	1,089	−186	−344	−29	0.021

Values are presented as absolute mean values or mean changes and 95% confidence interval (CI) with lower and upper limits at inclusion (baseline) (*n* = 30), 12 weeks after diet intervention(*n* = 30), and 16 weeks after the end of diet intervention (week 28) (*n* = 23). CCK = cholecystokinin, GLP-1 = glucagon-like peptide-1, GIP = glucose-dependent insulinotropic polypeptide, PAI-1 = plasminogen activator inhibitor-1, PYY = polypeptide YY. Linear mixed model. Comparisons were made between baseline and week 12 and week 28. *P* < 0.05 was considered statistically significant.

### Partial correlations between changes in hormonal levels and anthropometric and metabolic parameters

**Fig. 1:**
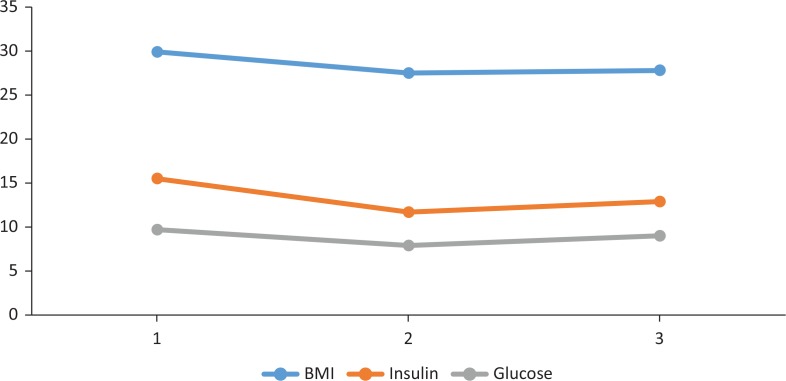
Mean values of body mass index (BMI) (kg/m^2^) and fasting levels of plasma glucose (mmol/L) and serum insulin (mIU/L) at baseline (1), week 12 (2) and week 28 (3) of the Okinawan-Based Nordic diet intervention in type 2 diabetes.

The endocrine changes were in alignment with changes of anthropometric and metabolic parameters (Fig. 1). Changes in concentrations of C-peptide and insulin correlated with changes in values of fasting glucose (*r_s_* = 0.474, *P* < 0.001 vs. *r_s_* = 0.344, *P* = 0.002), triglycerides (*r_s_* = 0.652, *P* < 0.001 vs. *r_s_* = 0.413, *P* < 0.001), and BMI (*r_s_* = 0.424, *P* < 0.001 vs. *r_s_* = 0.394, *P* < 0.001), and correlated inversely with changes in levels of HDL (*r_s_* = −0.509, *P* < 0.001 vs. *r_s_* = -0.301, *P* = 0.006), calculated by partial Spearman´s correlation test. In addition, the changes in C-peptide levels correlated with changes in HbA1c values (*r_s_* = 0.315, *P* = 0.004), and the changes in insulin levels correlated with changes in waist circumference (*r_s_* = 0.340, *P* = 0.006). Changes in glucagon and GLP-1 levels both correlated inversely with changes in LDL levels, whereas changed GIP levels correlated with changed HbA1c values. Changes in BMI and waist circumference correlated inversely with changes in ghrelin concentrations, and positively with changes in leptin concentrations, the latter also being correlated with changes in insulin resistance. Changes in PAI-1 and PYY levels correlated with changes in glucose and triglyceride levels, and PYY levels also correlated with changes of HbA1c values. Changes in resistin levels correlated with changes in systolic blood pressure (Table 4). There was no correlation between changes in CCK, cortisol, or visfatin levels and any calculated parameters (data not shown).

**Table 4:** Partial correlations between hormonal, metabolic, and anthropometric changes in type 2 diabetes before and after a 12-week Okinawa-based Nordic diet intervention

	Correlation coefficient	*P*
C-peptide		
Glucose	0.474	<0.001
HbA1c	0.315	0.004
High-density lipoprotein	−0.509	<0.001
Triglycerides	0.652	<0.001
Body mass index	0.424	<0.001
Insulin		
Glucose	0.344	0.002
High-density lipoprotein	−0.301	0.006
Triglycerides	0.413	<0.001
Body mass index	0.394	<0.001
Waist circumference	0.340	0.006
Glucagon		
Low-density lipoprotein	−0.324	0.003
GLP-1		
Low-density lipoprotein	−0.387	0.004
GIP		
HbA1c	0.335	0.002
Ghrelin		
Body weight	−0.482	<0.001
Body mass index	−0.444	<0.001
Waist circumference	−0.436	<0.001
Leptin		
Body mass index	0.507	<0.001
Waist circumference	0.465	<0.001
Insulin resistance	0.330	0.003
PAI-1		
Glucose	0.289	0.009
Triglycerides	0.309	0.005
Polypeptide YY		
Glucose	0.431	<0.001
HbA1c	0.356	0.001
Triglycerides	0.367	<0.001
Resistin		
Systolic blood pressure	0.297	0.008

GLP-1 = glucagon-like peptide-1, GIP = glucose-dependent insulinotropic polypeptide, PAI-1 = plasminogen activator inhibitor-1, PYY = polypeptide YY. Partial correlations, with the three observational time points, baseline, week 12, and week 28 from each individual, by Spearman´s correlation test. *P* < 0.01 was considered statistically significant.

## Discussion

Based on the study results presented here, we accepted the research hypothesis that intervention with the Okinawa-based Nordic diet affects several hormones released from the gut and pancreas with lower circulating levels of CCK, C-peptide, glucagon, GLP-1, GIP, insulin, and PYY. The concentrations of cortisol and leptin were reduced, and PAI-1 non-significantly reduced, after 12 weeks, whereas visfatin levels were reduced and resistin non-significantly reduced, after 28 weeks. Apart from CCK, cortisol, and visfatin levels, changes in hormonal levels correlated with the improved anthropometric and metabolic parameters. Despite reductions of weight, BMI, and waist circumference, the ghrelin levels were reduced after 28 weeks, compared to baseline.

The majority of the metabolic and anthropometric changes could be correlated with the endocrine profile, as expected from the literature (5, 6, 11, 12, 14, 16). The endocrine changes may be induced by the diet *per se*, secondary to the metabolic changes, or secondary to the weight reduction. After a single Okinawa-based Nordic breakfast to healthy subjects, the postprandial response of glucose, C-peptide, insulin, and GIP were attenuated compared with a traditional breakfast (4). The strongest correlations between anthropometric and metabolic parameters and hormone levels in the present study were observed with C-peptide and insulin levels, which suggest that the immediate endocrine response to the diet is crucial for the evolution of further effects. This hypothesis is strengthened by the fact that normalization of blood glucose *per se* did not affect postprandial levels of GLP-1 and GIP (23).

Both orexigenic (ghrelin) and anorexigenic hormone levels (CCK, glucagon, GLP-1, insulin, leptin, and PYY) were decreased in the present study. The overall decrease of hormonal levels reflects a balance in between the hormones; a decrease in one hormone level initiates a process of further influences. A reduction of GLP-1 and endogenous insulin production, as measured by reduced C-peptide levels, may depend on the reduced carbohydrate content and higher protein content in the diet (24), whereas the reduction in glucagon may be ascribed to the introduction of regular meals with shorter meal intervals, thereby improving substrate oxidation beneficially (25). Although the diet had a relatively high fat and protein content, the concentration of CCK and PYY were reduced after the dietary intervention. However, the study participant’s ordinary food may have had higher total amount of fat and protein than the Okinawa-based Nordic diet.

Although reductions of weight, waist circumference, and blood pressure remained at 28 weeks after study start, the hormonal levels, apart from PYY, had normalized. Noteworthy, ghrelin levels were reduced at follow-up. This is very interesting, since a previous study with a low-energy diet showed that the induced hormonal changes to increase sensation of hunger and encourage weight regain persisted until 1 year later, with elevated levels of ghrelin, which can explain the difficulties to maintain weight loss (26). Lower ghrelin levels render reduced appetite and less hunger (27). By changing the nutritional composition in the Okinawa-based Nordic diet, and not only reducing caloric intake, our participants exhibited lower ghrelin levels and increased or equal satiety with a good ability to maintain the weight loss (4, 28). This delayed effect on ghrelin may suggest a readjustment of appetite regulation and food intake after introduction of a healthier diet. The participants partly withheld the diet between 12 and 28 weeks, which may have contributed to a new balance in the hormonal system. This further suggests that dietary components have a huge influence on the endocrine control, and that the present endocrine alterations do not only depend on weight reduction *per se*. Previous research supports nutrient-specific effects on ghrelin secretion, which may counteract the elevated levels observed after weight loss in other studies (29). However, there may be synergistic effects between a healthy diet, improved metabolism, and weight reduction, but exact mechanisms are difficult to separate. Still, the most important from a clinical point of view is that introduction of a modified diet is correlated with a healthier endocrine profile. The weight loss of 0.5 kg/week was in the same magnitude as observed in previous, similar studies (30).

GIP has specific anabolic effects and enhances insulin secretion and insulin stimulation, which promotes accumulation of fat in adipose tissue (31). It is considered important to diminish the exaggerated GIP secretion, which demands altered chemical and physical food composition, as well as altered eating behaviors (32–34). The present results are in line with the anticipation that an overactive entero-insular axis may play a role in the development of diabetes and obesity (32).

Circulating adipokine levels are increased in obese subjects. Leptin and insulin show a strong relationship, and leptin resistance is accompanied by hyperinsulinemia and insulin resistance (14). The observed reduction of insulin concentration and insulin resistance by the Okinawa-based Nordic diet seem to be interconnected with lower levels of adipokines, although the close relation between resistin and insulin resistance found in other studies could not be confirmed in the present study (14). Resistin levels are elevated in hypertension (35), and, thus, the parallel decrease of systolic blood pressure and resistin in the present study is in alignment with this. Changes in cortisol levels may result from a multitude of factors including both diet (36) and stress in daily life (17).

It remains to determine which of the ingredients in the food are responsible for the observed hormonal and metabolic responses. The physical properties of the food were altered through higher fiber intake demanding more mastication, slower eating, greater gastric volume, and delayed gastric emptying rate. The chemical characteristics were changed with reduction of GI and processed food, lower energy percentage intake of carbohydrates, and higher energy percentage intake of fat and protein. In addition, most subjects had irregular meal habits at inclusion. Only a reduction of meal frequencies, without changes in the nutrition composition, may have great impact on glucose and hormone levels (37). The Ma-Pi 2 macrobiotic diet has similarities with the Okinawa-based diet regarding a high amount of whole grains, vegetables, and legumes, and no added sugar. In contrast, the daily energy percentages consist of 70 E% carbohydrate, 18 E% fat, and 12 E% protein (38). Still, these two diets have similar effects on type 2 diabetes (3, 38), which support previous suggestion that whole-grain cereals, high fiber intake, and unprocessed food is more important than the relative amounts of nutrients (39, 40). Fibers and whole grains act as prebiotics (41), which has been shown to increase PYY and GLP-1 secretion (38), in contrast to our findings. In epidemiologic studies, mortality was lower in subjects with a high intake of whole-grain products, especially intake of breakfast cereals and nonwhite bread (42). Regular consumption of breakfast cereals, especially whole grains, is associated with less overweight and development and management of diabetes (43). The association between high intake of whole-grain products and overall healthier lifestyle habits may reduce the associations to be markers of healthy habits (44). In the present prospective study, the positive effects by dietary modification were observed without concomitant intervention of other lifestyle factors and independently of socioeconomic factors.

One of the limitations of the present study was that only fasting hormonal levels were analyzed and not the total area under the curve during the day. Varying degrees of diabetic complications may also affect results. In the present study, only one patient had verified gastroparesis, which should not influence the result. More patients suffered from autonomic neuropathy, which is, however, not associated with altered concentration of gut hormones in type 1 diabetes (45). Another limitation is the absence of an external control group, which provides some evidence that changes occurring over time were not the result of natural temporal trends or of unmeasured events that occurred contemporaneously with the study. It is a challenge to construct an appropriate control group in open, nonblinded lifestyle studies. A similar dietary interventional study using a control group advised to follow their habitual diet and physical activity did however not show any changes in the controls during the observational time (30). Instead, the participants were characterized at baseline and followed during and after dietary intervention, thus being their own controls to measure intra-individual differences. When people anticipate eating a scheduled meal, cephalic responses induce an increased secretion of several meal-related hormones before the start of the meal (46). Thus, the very expectancy of entering a lifestyle change should have altered the hormonal profile already at baseline. The alterations found thereafter should be secondary to dietary changes, metabolic alterations, and/or weight reductions.

In conclusion, a 12-week dietary intervention in type 2 diabetes with an Okinawa-based Nordic diet has significant impact on the endocrine profile, which is in alignment with the anthropometric and metabolic improvements.

## Supplementary Material

Alignments of endocrine, anthropometric, and metabolic parameters in type 2 diabetes after intervention with an Okinawa-based Nordic diet
