# Supplementary material for: Alignments of endocrine, anthropometric, and metabolic parameters in type 2 diabetes after intervention with an Okinawa-based Nordic diet
Source: Food Nutr Res. 2018 Mar 14;62:10.29219/fnr.v62.1328. doi: 10.29219/fnr.v62.1328 (PMC5854836; doi:10.29219/fnr.v62.1328)
Supplement: Alignments of endocrine, anthropometric, and metabolic parameters in type 2 diabetes after intervention with an Okinawa-based Nordic diet [file FNR-62-1328-s001.pdf]

Supplementary Table 1. Nutrition composition of the Okinawan-based Nordic diet

**Nutritional value**	**Unit**	**Calculated value**	**E%**	**Recommended (NNR 2012)**
Total Energy	kcal	1866		
Energy (excluding beverages)	kcal	1629		
Protein	g	95.0	23	10–20 E%
Fat	g	63.9	35	25–40 E%
Saturated fatty acids	g	18.7	10	<10 E%
Polyunsaturated fatty acids	g	14.9	8	5–10 E%
Mono-unsaturated fatty acids	g	17.8	10	10–20 E%
Carbohydrate	g	168.4	42	45–60 E%
Sucrose	g	23.5	6	<10 E%
Dietary fibre	g	35.9		25–35 g

E% = Energy percentage. Daily mean intake of energy, nutrients, and food components of the modified Okinawan-based Nordic diet, compared with Nordic Nutrition Recommendations (NNR) 2012 (ref No 18).

Supplementary Table 2. Inter-assay coefficient of variation (cv) for controls in Luminex analyses

Diabetic biomarker	Low control inter-assay cv % (n)	High control inter-assay cv % (n)	Intra-assay cv* %
C-peptide	17.0 (7)	4.9 (7)	5
Ghrelin	18.0 (8)	12.2 (8)	4
Glucagon	16.4 (8)	13.5 (8)	8
GLP-1	10.5 (8)	13.9 (8)	8
GIP	8.3 (8)	9.9 (8)	3
Insulin	9.0 (8)	8.2 (8)	3
Leptin	8.7 (8)	6.7 (8)	3
PAI-1	7.4 (5)	12.8 (4)	5
Resistin	4.3 (8)	10.5 (8)	3
Visfatin	29.0 (8)	15.5 (8)	4

*= Intra-assay coefficient of variation (cv) % is according to Bio-Rad Laboratories. n = number of samples. GLP-1 = Glucagon-like peptide-1, GIP = Glucose-dependent insulinotropic polypeptide, PAI-1 = plasminogen-activator inhibitor-1.

Supplemental Figure 1. Flow-chart of patient recruitment and study design. Two patients interrupted the study at 6 weeks on diet, and data collected at that time point was calculated together with data from patients with 12 weeks of intervention.

Recruitment of patients with type 2 diabetes at a primary health care center

Inclusion criteria:
Age span: 18–70 years
Both parents born in Scandinavia
Type 2 diabetes

Exclusion criteria:
Inability to understand the Swedish language
Severe food allergy
Severe heart, pulmonary, cardiovascular, malignant or psychiatric diseases
Severe liver disease (spontaneous prothrombin complex (INR) > 1.1)
Severe renal disease (glomerular filtration rate (GFR) < 30 mL/min/1.73 m^2)
Pregnancy
Already on ongoing weight-reducing diet
Major prior gastrointestinal surgery
Alcohol and/or drug abuse

45 available patients contacted by mail and phone

15 patients excluded or unwilling to participate:

Unwillingness (n = 11)
Late autoimmune diabetes in adult (n = 1)
Gastric by-pass surgery (n = 1)
Pregnancy (n = 1)
Already on another diet (n = 1)

30 patients included at baseline
Anthropometry
Blood sampling
Study and nutrition questionnaires
Study protocol

30 patients examined at week 2 and 6 after dietary intervention
Anthropometry
Study protocol

28 patients examined at week 12 and 2 patients at week 6 after dietary intervention
Anthropometry
Blood sampling
Study and nutrition questionnaires
Study protocol

2 drop outs at week 6:

Work-related time constraints (n = 1)
Family-related problems (n = 1)

23 patients at 28-week follow-up
Anthropometry
Blood sampling
Study and nutrition questionnaires
Study protocol

5 drop outs at week 28:

Unwillingness to continue (n = 2)
Depression (n = 2)
Family-related problems (n = 1)
